# Immunomodulatory potential of anti-IFN-beta antibodies on monocyte-derived macrophages

**DOI:** 10.5339/qmj.2023.sqac.13

**Published:** 2023-11-19

**Authors:** Ajith Sominanda, Til Menge, Hans Peter Hartung, Bernd C Kieseier

**Affiliations:** ^1^College of Medicine, Department of Basic Medical Sciences, Qatar University, Doha, Qatar Email: ajithsomi@qu.edu.qa; ^2^Department of Neurology, Heinrich Heine University, Düsseldorf, Germany

## Abstract

**Introduction:** Multiple sclerosis (MS) is a disabling neurological disease with an unknown etiology, where the recombinant interferon beta (rIFNβ) is the most established treatment. However, the development of anti-IFNβ antibodies has posed a significant therapeutic drawback. In this study, the interaction between anti-IFNβ antibodies and macrophages was investigated to assess the effects on the immune system.

**Methodology:** Using magnetic beads, anti-IFNβ antibodies were extracted from MS patients’ sera positive for anti-IFNβ antibodies. A negative control (antibody-negative situation) and a baseline control were obtained in parallel. Bead or extracted beadantibody complexes were then incubated *vitro* with monocyte-derived human macrophages. After incubation, macrophage cultures were tested for 91 immunologically relevant gene expressions by RT-PCR.

**Results and Discussions:** A Gene expression difference between antibody positive and negative situations was hypothesized to reflect the direct effects between antibodies and macrophages. Thus, 37-39 genes were either up-regulated or downregulated due to this direct interaction. Of these, only 2-4 genes were up-regulated, and the rest were down-regulated. These observations suggest that anti-IFNβ antibodies have an overall suppressive effect on immunologically relevant gene activity when antibodies interact with macrophages.

**Conclusion:** The fate and effects of circulating anti-IFNβ antibodies are mainly unknown. With the observations obtained at *in vitro* level, such effects, especially from an immunological point of view, are suppressive on immunocompetent cells such as macrophages. However, *in vivo* verification is necessary.

